# HER2 Oncogenic Function Escapes EGFR Tyrosine Kinase Inhibitors via Activation of Alternative HER Receptors in Breast Cancer Cells

**DOI:** 10.1371/journal.pone.0002881

**Published:** 2008-08-06

**Authors:** Anthony Kong, Véronique Calleja, Pierre Leboucher, Adrian Harris, Peter J. Parker, Banafshé Larijani

**Affiliations:** 1 Cell Biophysics Laboratory, Lincoln's Inn Fields laboratories, London Research Institute, Cancer Research UK, London, United Kingdom; 2 Protein Phosphorylation Laboratory, Lincoln's Inn Fields laboratories, London Research Institute, Cancer Research UK, London, United Kingdom; 3 Weatherall Institute of Molecular Medicine (IMM), Cancer Research UK, University of Oxford, John Radcliffe Hospital, Oxford, United Kingdom; 4 Laboratoire de Physiologie de la Perception et de l'Action, College de France, Paris, France; 5 Division of Cancer Studies, King's College School of Medicine, Guy's Hospital, London, United Kingdom; Ordway Research Institute, United States of America

## Abstract

**Background:**

The response rate to EGFR tyrosine kinase inhibitors (TKIs) may be poor and unpredictable in cancer patients with EGFR expression itself being an inadequate response indicator. There is limited understanding of the mechanisms underlying this resistance. Furthermore, although TKIs suppress the growth of HER2-overexpressing breast tumor cells, they do not fully inhibit HER2 oncogenic function at physiological doses.

**Methodology and Principal Findings:**

Here we have provided a molecular mechanism of how HER2 oncogenic function escapes TKIs' inhibition via alternative HER receptor activation as a result of autocrine ligand release. Using both Förster Resonance Energy Transfer (FRET) which monitors *in situ* HER receptor phosphorylation as well as classical biochemical analysis, we have shown that the specific tyrosine kinase inhibitors (TKIs) of EGFR, AG1478 and Iressa (Gefitinib) decreased EGFR and HER3 phosphorylation through the inhibition of EGFR/HER3 dimerization. Consequent to this, we demonstrate that cleavage of HER4 and dimerization of HER4/HER2 occur together with reactivation of HER3 via HER2/HER3, leading to persistent HER2 phosphorylation in the now resistant, surviving cells. These drug treatment–induced processes were found to be mediated by the release of ligands including heregulin and betacellulin that activate HER3 and HER4 via HER2. Whereas an anti-betacellulin antibody in combination with Iressa increased the anti-proliferative effect in resistant cells, ligands such as heregulin and betacellulin rendered sensitive SKBR3 cells resistant to Iressa.

**Conclusions and Significance:**

These results demonstrate the role of drug-induced autocrine events leading to the activation of alternative HER receptors in maintaining HER2 phosphorylation and in mediating resistance to EGFR tyrosine kinase inhibitors (TKIs) in breast cancer cells, and hence specify treatment opportunities to overcome resistance in patients.

## Introduction

The human Epidermal Growth Factor Receptor (HER, also known as ErbB) family consists of four receptors EGFR (HER1 or ErbB-1), HER2 (ErbB-2), HER3 (ErbB-3) and HER4 (ErbB-4) binding more than 10 polypeptide ligands between them [1]. The HER receptors play a crucial role in breast cancer and many other types of cancer [2], generating much interest in understanding their individual and combinatorial actions. These receptors belong to subclass I of the superfamily of Receptor Tyrosine Kinases (RTKs) which are transmembrane receptors with an intrinsic ability to phosphorylate their tyrosine residues in the cytoplasmic domains to transduce signals [3]. However, HER2 and HER3 are not autonomous since HER2 has no known ligand and the kinase activity of HER3 is defective [2]. These two receptors can form heterodimeric complexes with each other as well as other HER receptors to generate potent signals [4].

The response rate to EGFR or HER2 inhibitor monotherapy remains very poor despite a selection of patients based on EGFR or HER2 over-expression [5], [6]. In addition, the expression of HER receptors does not seem to predict the response to these drugs [7], [8]. Patients with EGFR mutations respond extremely well to Iressa [9] but these are only found in a small subset of patients [10]. Therefore, the underlying mechanisms contributing to the resistance as well as predicting the success of these drugs in cancer patients are still poorly understood. The response rate to targeted HER family therapy depends on more than just the receptor concentrations or the mutations of the particular HER receptor. It is likely that multiple interacting HER receptors and ligands are involved in mediating the response to targeted therapy. For example EGFR tyrosine kinase inhibitor (TKI) like Iressa (Gefitinib, ZD 1839) which targets the EGFR receptor also inhibits the PI3K and PKB pathway via HER3 [11]. Moreover, Iressa is also effective in HER2 over-expressing breast cancer cells [12]. Therefore, treatment that reduces the tyrosine kinase activity of EGFR receptors may also affect HER2 and HER3 receptors. It has been argued that therapy based on receptor concentration, ignoring the activation and phosphorylation state of the receptor and its interaction with other HER receptors continues to yield a relatively low response rate [13], [14].

Targeting HER2 has been the main focus in breast cancer 15, 16, 17 although increasingly, inhibition of EGFR in combination with HER2 blockage is seen to be important in breast cancer therapy. Moreover, EGFR expression had also been shown to play a role in hormone resistant breast cancer patients [18] and this has led to the use of Iressa with aromatase inhibitors in breast cancer [19]. More recently Lapatinib which targets the tyrosine kinase activities of both EGFR and HER2 has been shown to be beneficial in HER2 positive patients, confirming the important role of EGFR inhibition in breast cancer [20].

HER2 phosphorylation maybe used as a surrogate marker for the activation status of other HER receptors, being the preferred dimerization partner [21]. Therefore, the main aim of the study was to assess the effects of TKIs on changes in HER2 phosphorylation status in relation to other HER receptors in breast cancer cell lines. TKIs had been shown to inhibit HER2-driven signaling and to suppress the growth of HER2-overexpressing breast tumor cells [22], [23]. However, it was also reported that TKIs do not fully inhibit HER2 oncogenic function at conventional doses and concentrations [24]. To resolve the controversy, we used FRET to study activation changes in HER2 and other HER receptors in relationship to TKIs treatment in breast cancer cell lines. FRET can detect HER2 phosphorylation variations with greater sensitivity than classical biochemical methods. Further, analysis of single cells by FRET provides information inaccessible through conventional biochemistry. We demonstrated that the HER2 phosphorylation was not fully inhibited by TKIs in the surviving cells due to the activation of alternative HER receptors through their ligands. These mechanisms may mediate resistance to the TKIs in breast cancer cell lines. The combined treatment of cells with Herceptin (Trastuzumab) and Iressa exerted a greater suppression on EGFR and HER2 phosphorylation, and induced an enhanced anti-proliferative effect. Our data provides evidence that therapy based on the assessment of engagement of all four EGF receptors should improve outcomes.

## Results

We applied FRET to study the effect of TKIs on HER2 phosphorylation since FRET can detect variations between single cells not accessible through other biochemical methods. Having previously established the assessment of EGFR phosphorylation state by Förster Resonance Energy Transfer (FRET) in A431 cells [14], we applied FRET to assess HER2 phosphorylation in relation to TKIs in our test cell line A431 cells as well as various breast cell lines with variable HER2 expression.

### HER2 phosphorylation state monitored by FRET

HER2 is not known to have its own ligand although it dimerizes with other HER receptors via their respective ligands [21]. To establish an assay for HER2 phosphorylation state, it was necessary to trigger HER2 phosphorylation via other HER receptors. We chose A431 cells as a test cell line because of their extensive prior use for the analysis of EGFR and other HER receptors. EGFR and HER2 levels in relation to three breast cell lines (MCF-7, MDAMB-453, SKBR3) are illustrated in Figure S1A.

We conjugated anti-HER2 antibody to a Cy3b chromophore (HER2-Cy3b) and an anti-phosphoHER2 antibody to Cy5 (pHER2-Cy5) to assess HER2 phosphorylation in fixed cell samples (see Methods). The hypothesis was that upon HER2 activation there would be phosphorylation of the receptor and therefore FRET between the two bound antibodies. The consequent specific quenching of the donor chromophore Cy3b would result in the decrease of lifetime of HER2-Cy3b and therefore the decrease of lifetime of HER2-Cy3b is indicative of HER2 phosphorylation status (see Methods).

**Figure 1:**
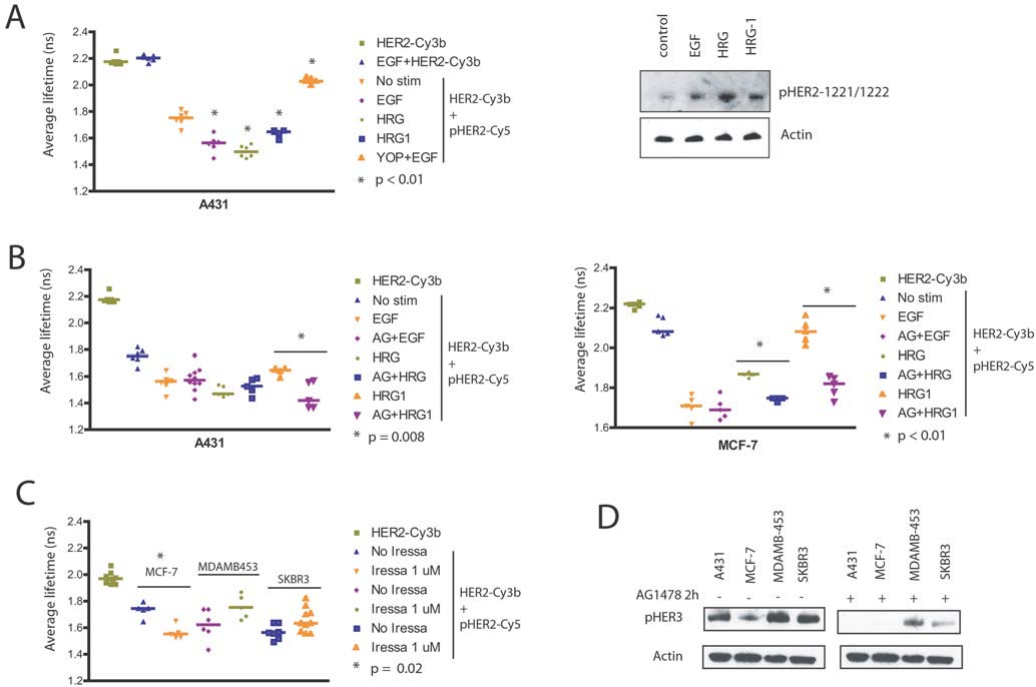
Inhibition of EGFR with AG 1478 and Iressa does not abolish HER2 phosphorylation. A, Displayed is the average lifetime of HER2-Cy3b in A431 cells treated under different conditions as indicated. Each point represents one measurement of the average lifetime of HER2-Cy3b in A431 cells (single cell or group of cells in a single field) with the lines representing the median average lifetime of all the cells under each condition. To assess HER2 activation in A431 cells by FRET, we incubated the cells with either donor alone (HER2-Cy3b) or donor and acceptor (HER2-Cy3b+pHER2-Cy5) after 10 minutes stimulation with either 100 ng/ml EGF, 100 ng/ml heregulin β (HRG) or 100 ng/ml heregulin β-1 (HRG1). To remove phosphotyrosine, the phosphatase YOP was used following stimulation of the cells with EGF. On the right panels, near confluent A431 cells were stimulated with EGF, heregulin β and heregulin β-1 for 10 minutes. 10 µg of protein was used for western blot analysis. The phosphorylation of HER2 on Tyr1221/1222 was determined with a phosphospecific antibody. B, A431 cells (left graph) were pre-treated with 3 µM of the tyrosine kinase inhibitor AG 1478 (AG) for two hours before stimulation with either EGF, heregulin β or heregulin β-1 as indicated. The average lifetime of HER2-Cy3b for those cells pre-treated with AG 1478 was compared with those without treatment using the Mann-Whitney test. The same experiment was also performed in MCF-7 cells (right graph). C, MCF-7, MDAMB-453 and SKBR3 cells were pre-treated with 1 µM Iressa for 2.5 days before assessing their HER2 phosphorylation as above. D, A431 cells, MCF-7, MDMAB-453 and SKBR3 were pre-treated with 1 µM AG 1478 for two hours. The cells were treated with lysis buffer and proteins separated by SDS-PAGE. The phosphorylation of HER3 on Tyr1289 was determined using an anti-phosphospecific antibody.

To show *in-situ* that HER2 could be activated upon dimerization with other members of the HER family, A431 cells were stimulated with EGF, heregulin β and heregulin β-1 (ligands for HER1, HER3 and HER4 respectively). The average lifetime of the donor HER2-Cy3b alone (detecting HER2 protein) was 2.20 ns (Figure 1A) and EGF stimulation alone in the absence of acceptor-coupled second antibody did not affect the donor lifetime. In the presence of the acceptor antibody pHER2-Cy5 (detecting phosphorylated HER2), the donor lifetime of HER2-Cy3b decreased to 1.75 ns due to basal HER2 phosphorylation (Methods - Interpretation of FRET Data). Further significant decreases in the average lifetime of HER2-Cy3b were measured upon EGF, β and β-1 heregulin stimulation (Figure 1A). The significant decreases in average lifetime compared to the basal level (p<0.01) indicate an increase in HER2 tyrosine phosphorylation and therefore activation in A431 cells. To verify the measurements were not due to non-specific FRET, the phosphatase YOP was used after EGF treatment to dephosphorylate phosphotyrosine residues on HER2. The average lifetime reversed to the control values (orange triangles) indicating a loss of FRET. In parallel an increase in HER2 phosphorylation on Tyr1221 and 1222 in a total cell lysate was shown by western blot using a phospho-specific antibody (Figure 1A, right panels). Moreover, heregulin β and β-1 did not induce EGFR activation in A431 cells (data not shown).

Together these data indicated that *in-situ* HER2 phosphorylation by ligands of other HER receptor family members could be monitored by FRET.

### The effect of tyrosine kinase inhibitors of EGFR on HER2 activation states

As HER2 is the preferred dimerization partner for EGFR and other HER receptors, we proceeded to determine the effect of TKIs on HER2 phosphorylation state induced through other HER receptors under various conditions. Since A431 cells over-express EGFR, we expected AG 1478 to prevent activation of HER2 by EGF stimulation. However, AG 1478 failed to abolish EGF-induced HER2 phosphorylation in A431 cells (Figure 1B). Heregulin β induced HER2 phosphorylation was also not inhibited by AG1478. AG1478 increased HER2 phosphorylation in the presence of heregulin β-1, indicated by a decrease of average donor lifetime compared to heregulin β-1 alone (p = 0.008) in A431 cells (Figure 1B, left graph). In MCF-7 cells, AG 1478 also did not abolish EGF induced HER2 phosphorylation. Phosphorylation of HER2 was greater by heregulin β and heregulin β-1 in the presence of AG 1478 (p<0.01 for both conditions; Figure 1B, right graph). Increased doses of acute AG 1478 treatment up to 300 µM failed to abolish EGF induced HER2 phosphorylation in A431 cells (Figure S1B), despite its effect on PKB and ERK1/2 phosphorylation (Figure S1C). The inability of AG 1478 to abolish HER2 phosphorylation was not due to EGF stimulation since treatment of AG 1478 alone without EGF stimulation also failed to abolish HER2 phosphorylation in A431 cells and two other breast cancer lines, MDAMB-453 and SKBR3 (Figure S1D, upper panels) despite the effect on PKB and ERK 1/2 phosphorylation (Figure S1D, lower panels). We proceeded to investigate whether Iressa, another more potent EGFR TKI had the same effect on HER2 phosphorylation in various breast cells. Figure 1C shows that acute treatment with 1 µM Iressa did not abolish basal HER2 phosphorylation in MCF-7 cells but induced a significant increase in its phosphorylation, resulting in a further decrease of lifetime (p = 0.02 compared to basal lifetime). In HER2 over-expressing MDAMB-453 and SKBR3, some cells show partial HER2 phosphorylation but overall HER2 phosphorylation was not abolished (Figure 1C).

Although TKIs induce the formation of inactive EGFR/HER2 [22], [23], we showed that they failed to abolish basal HER2 phosphorylation. This suggested that the persistence of HER2 activation was not be due to EGFR/HER2 dimerization, but from either HER3/HER2 or HER4/HER2 dimerization. We also showed that the EGFR inhibition potentiated HER2 phosphorylation by exogenous heregulin stimulation, suggesting that HER3/HER2 and HER4/HER2 dimers could occur to sustain HER2 phosphorylation. However, TKIs including AG 1478 and Iressa decreased HER3 phosphorylation (Figure 1D). Therefore, the increased HER2 phosphorylation upon heregulin stimulation with TKI treatment (Figure 1B) indicated the involvement of HER4 in sustaining HER2 phosphorylation.

### AG 1478 and Iressa induce proteolytic cleavage of HER4 as well as dimerization between HER2 and HER4 in breast cancer cell lines

It has been shown that proteolytic cleavage of HER4 occurs in cells at a low basal level and can be increased by heregulin, or other growth factors that bind to HER4 [25]. The ectodomain cleavage of HER4 is mediated by tumour necrosis factor-α-converting enzyme (TACE), a transmembrane metalloproteinase that produces a membrane-anchored fragment (80 kD) which consists of the entire cytoplasmic and transmembrane domain [26], [27]. The m80 HER4 fragment from ectodomain cleavage was found to associate with full length HER2 [28]. In addition, the transmembrane m80 was found to be cleaved by γ-secretase and the soluble fraction (S80) was found to be translocated to the nucleus [29], [30]. The cleaved HER4 fragment remains phosphorylated in the membrane, cytoplasmic and nuclear extracts following heregulin stimulation [31], suggesting that the cleaved fragment may be used as a reporter for HER4 activation.

**Figure 2:**
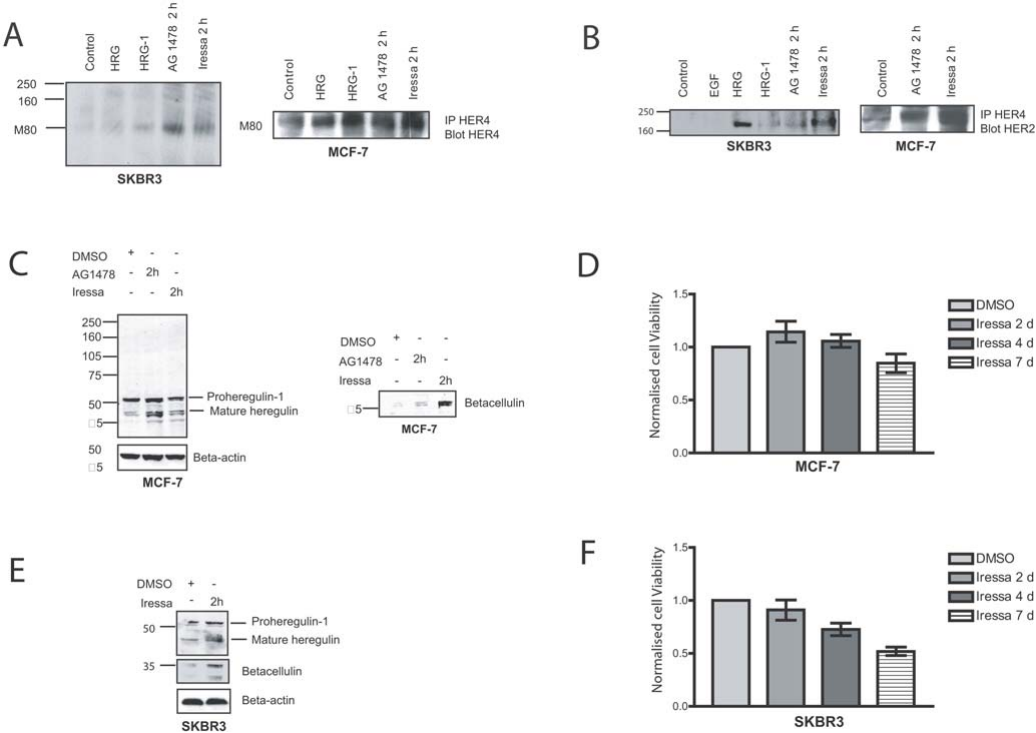
AG 1478 and Iressa induce proteolytic cleavage of HER4 and dimerization between HER2 and HER4 in breast cancer cell lines via the autocrine ligand release. A, HER4 was immunoprecipitated from SKBR3 and MCF-7 cells after being treated with the conditions illustrated. Following the immunoprecipitation, the cell lysate was probed by western analysis for total HER4. B, HER4 was immunoprecipitated from both SKBR3 and MCF-7 cell lysates after treatment under the conditions illustrated. Extracts were probed with anti-HER2 antibody. C, MCF-7 cells were treated with either 3 µM AG 1478 or with 1 µM Iressa for 2 hours before the cells were lysed. 10 µg of protein was loaded in each lane for SDS-PAGE and the membrane was probed with an antibody recognizing proheregulin-1 or betacellulin. D MCF-7 cells were grown in 24-well plates after seeding around approximately 30,000 cells per well and left to grow for at least 24 hours before treatment with either DMSO or 1 µM Iressa for the different durations. The viable cells were counted in a cell viability analyzer using Trypan Blue to stain dead cells. E, Near confluent SKBR3 cells were pre-treated with Iressa or DMSO for 2 hours as illustrated before the cells were lysed for western blot experiments. The membrane was probed with antibodies recognizing proheregulin-1 and betacellulin with anti-β-actin antibody used as loading control. F, SKBR3 cells were grown in 24-well plates after seeding around approximately 30,000 cells per well. They were treated with either DMSO or 1 µM Iressa for the different durations and the viable cells were counted in a cell viability analyzer and normalised to the control.

We postulated that maintenance of HER2 activation and the enhanced HER2 phosphorylation by heregulin stimulation combined with AG 1478 may be due to activation of HER4 with the subsequent activation of HER2. We therefore assessed HER4 cleavage and its interaction with HER2 following EGFR inhibition by AG 1478 or Iressa. Figure 2A illustrates the cleavage of HER4 and production of m80 upon heregulin stimulation in SKBR3 and MCF-7 cells. Moreover, acute treatment with the tyrosine kinase inhibitor AG 1478 or Iressa also induced the cleavage of HER4 and production of m80 in both SKBR3 and MCF-7 cells (Figure 2A). Upon tyrosine kinase inhibition the m80 fragment accumulation was augmented compared to the response to exogenous heregulin. To prove further that the maintenance of HER2 phosphorylation was due to HER4 activation, we assessed the dimerization between HER2 and HER4. Indicative of dimerization in SKBR3 and MCF-7 cells, Figure 2B illustrates the co-immunoprecipitation of HER2 with intracellular anti-HER4, induced by heregulin stimulation or EGFR inhibition with either AG 1478 or Iressa.

Upon acute treatment with AG 1478 and Iressa, downstream signalling pathways are inhibited due to the prevention of EGFR homodimers and EGFR/HER2, EGFR/HER3 heterodimer formation, consistent with other reports [11], [23]. Nevertheless, proteolytic cleavage of HER4 and heterodimerization of HER2/HER4 occurred and thus sustained HER2 phosphorylation.

### AG 1478 and Iressa induce the release of ligands including heregulin and betacellulin

We showed above that acute treatment of AG 1478 and Iressa caused proteolytic cleavage of HER4 as well as dimerization of HER2/HER4, a response characteristic of heregulin stimulation. This suggested that tyrosine kinase inhibitors, which target EGFR, may trigger the release of ligands that induce HER4 cleavage.

Indeed we observed that AG 1478 and Iressa induced the cleavage of the precursor proheregulin-1 producing mature heregulin, which migrates between 35 and 50 kDa (Figure 2C, left panel). The most extensive cleavage of proheregulin-1 was seen with AG 1478 treatment although there was also an increase on Iressa treatment. The treatment with either drug also increased the production of betacellulin in MCF-7 cells (Figure 2C, right panel). In contrast to heregulin release, the maximum increase of betacellulin was seen with acute Iressa treatment rather than AG 1478 (Figure 2C, right panel). MCF-7 cells are generally considered to be resistant to physiological doses of Iressa. Using cell viability assays we confirmed that during acute treatment with 1 µM Iressa, MCF-7 growth was not prevented and furthermore there was an increase in cell proliferation compared to the control (Figure 2D). After seven days of treatment, MCF-7 cell growth was only minimally inhibited by 1 µM of Iressa (Figure 2D). SKBR3 cells are known to be sensitive to Iressa due to the inhibition of EGFR/HER2 and EGFR/HER3 [12], [32] and we have confirmed their sensitivity to Iressa using cell viability assays (Figure 2F). We have also shown that there was an increase in cleavage of pro-heregulin-1 as well as an increase in betacellulin production induced by two hours of Iressa treatment in sensitive SKBR3 cells (Figure 2E).

We have shown that the activation and proteolytic cleavage of HER4 occurred during acute treatment of EGFR tyrosine kinase inhibitors correlated with the release of ligands including betacellulin and heregulin in both resistant MCF-7 cells and sensitive SKBR3 cells.

### Prolonged Iressa treatment caused reactivation of HER3 activity in both resistant MCF-7 cells and sensitive SKBR3

**Figure 3:**
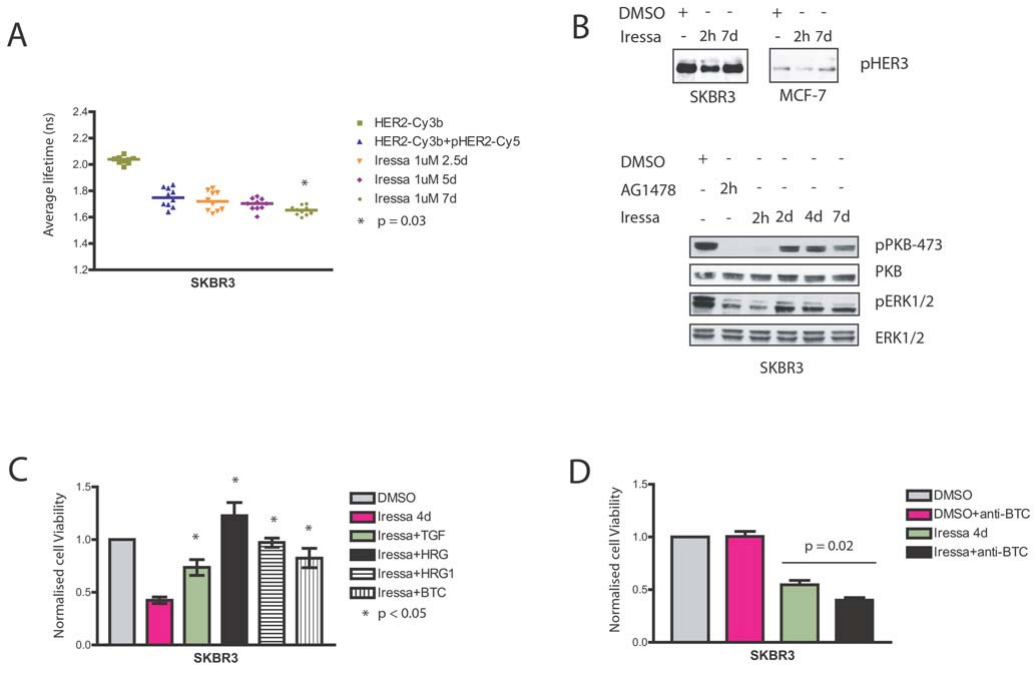
HER2 activation is maintained in surviving SKBR3 cells with reactivation of HER3 and downstream signalling pathways via the autocrine ligand release. A, SKBR3 cells were assessed for HER2 phosphorylation by FRET (see Methods) after the cells were treated with 1 µM Iressa for different durations. B, In the upper panel, SKBR3 and MCF-7 cells were lysed after treatment with either DMSO or 1 µM Iressa for the durations shown and the phosphorylation state of HER3 on Tyr1289 was determined using phosphospecific antibody. In the lower panels, SKBR3 cells were lysed after treatment with either DMSO, 3 µM AG 1478 or 1 µM Iressa for the durations illustrated. Phospho-PKB, phospho-MAPK and the total levels of PKB and Erk1/Erk2 were assessed using appropriate the antibodies. C, Cell viability experiments with SKBR3 cells were performed after treatment with DMSO or 1 µM Iressa with or without growth factors for 4 days. In the first condition DMSO was used as a vehicle control. In other conditions 1 µM Iressa was utilised alone or together with 100 ng/ml TGF, 100 ng/ml heregulin β, 100 ng/ml heregulin β-1 or 20 ng/ml betacellulin. The viable cells were counted in a cell viability analyzer after 4 days using Trypan Blue to stain dead cells. D, SKBR3 cells were treated with 20 µg/ml of anti-betacellulin, Iressa alone or Iressa in combination with 20 µg/ml of anti-betacellulin for 4 days before the cells were counted in a cell viability analyzer. DMSO was used as a vehicle control.

Iressa has been shown to inhibit the PI3K/PKB pathway via HER3 [11]. We observed a rapid decrease of phospho-HER3 (Figure 1D) and phospho-PKB (Ser473) upon acute treatment of AG1478 (Figure S1C and S1D) through inhibition of EGFR/HER3 [11], [23]. However, acute treatment of Iressa induced the release of heregulin in both MCF-7 and SKBR3 causing dimerization of HER2 and HER4 (Figure 2C and 2E). Since heregulin is the ligand for both HER3 and HER4, we considered that acute Iressa treatment may have induced dimerization of HER2/HER3 as well as HER2/HER4, maintaining HER2 activation. Figure 3A shows that seven days of Iressa treatment was not able to abolish HER2 phosphorylation even in sensitive SKBR3 (Figure 2F). After seven days of Iressa treatment, the remaining surviving cells had an enhanced HER2 phosphorylation monitored by FRET compared to basal conditions (p = 0.03) (Figure 3A). Moreover, not only was HER2 phosphorylation maintained in surviving SKBR3 cells (Figure 3A), but phospho-HER3 was reactivated with prolonged Iressa treatment (Figure 3B). The reactivation occurred after the initial decrease in HER3 activation (Figure 1D and 3B) via inhibition of EGFR/HER3 [11], [23] in both SKBR3 and MCF-7 cells. The reactivation was not due to the degradation of the drugs since the dose of Iressa was replenished after a few days. We also observed the recovery of phospho-PKB (Ser473) and phospho-ERK1/2 within 48 hours (Figure 3B, lower panels), consistent with activation of alternative HER pathways including HER2/HER3 and HER2/HER4 via autocrine release of ligands.

### The autocrine ligand release mediates resistance to Iressa in sensitive SKBR3 cells

To test the hypothesis that activation of alternative HER receptors through the autocrine release of ligands mediates resistance to Iressa, we stimulated sensitive SKBR3 cells with TGF-α, heregulin-β, heregulin β-1 or betacellulin while the cells were treated with Iressa for 4 days. Figure 3C shows that all the ligands rendered the sensitive SKBR3 resistant to Iressa. The greatest effect was seen with Iressa treatment in combination with either heregulin β or heregulin β-1. The results are consistent with previous experiments where EGFR inhibition by tyrosine kinase inhibitors sensitises the cells to exogenous heregulin stimulation in terms of HER2 activation (Figure 1B) and hence induced enhanced proliferation. This experiment confirms the role of ligands in mediating resistance to Iressa.

To test if the resistance of SKBR3 cells was accounted by the autocrine ligand release, a neutralising antibody was employed. An anti-betacellulin antibody (which blocks the effects of betacellulin) in combination with Iressa was found to potentiate the inhibitory effect of Iressa in cell viability experiments (Figure 3D). The results indicate a role of autocrine ligand release in mediating resistance to Iressa.

### Combined therapy with Herceptin and Iressa exerts a greater suppression in EGFR and HER2 activation

**Figure 4:**
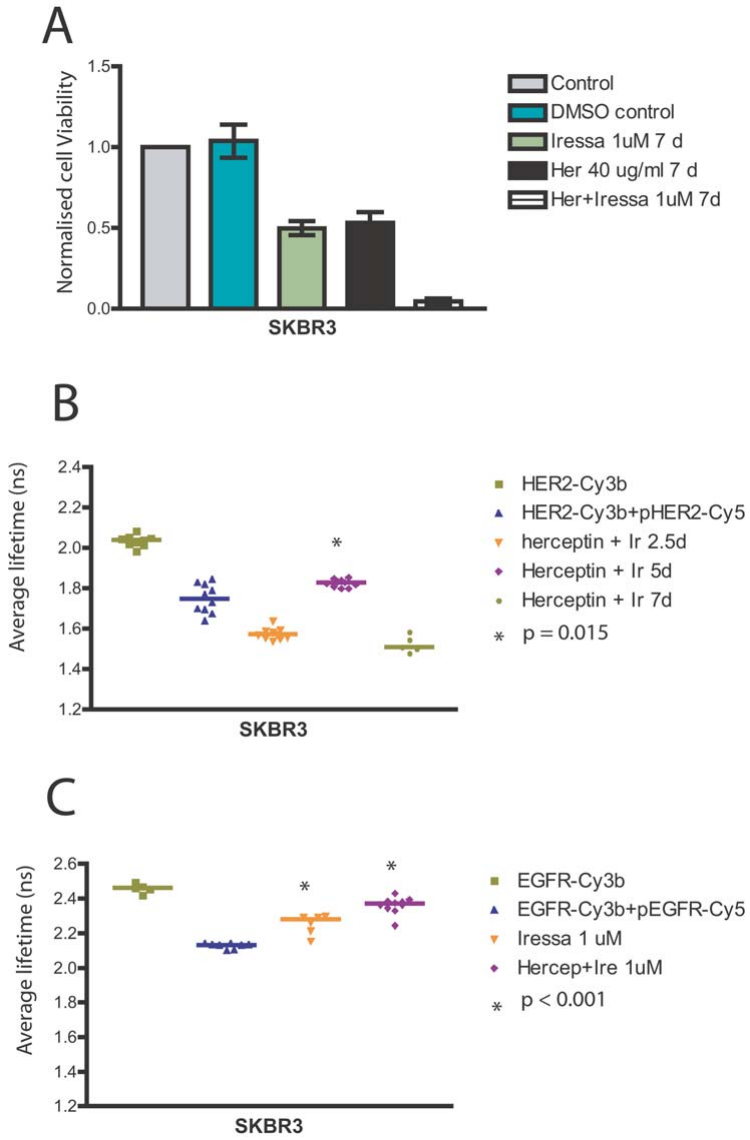
Combined therapy of Iressa and Herceptin was additive compared to either therapy alone due to greater inhibition of EGFR and HER2 phosphorylation. A, SKBR3 cells were grown in 24-well plates for at least 24 hours before treatment with 1 µM Iressa, 40 µg/ml Herceptin or 1 µM Iressa with 40 µg/ml Herceptin for 7 days. The viable cells were counted in a cell viability analyzer and normalised to the control. B, SKBR3 cells were treated with 40 µg/ml Herceptin and 1 µM Iressa for different durations and HER2 activation was monitored by FRET. A Mann-Whitney test was used to compare the medians of their lifetimes with the basal state (no drug treatment). C, SKBR3 cells were pre-treated with either 1 µM Iressa or combined treatment of 40 µg/ml Herceptin and 1 µM Iressa for 2.5 days before assessment of EGFR phosphorylation by FRET. The medians of the average lifetimes for the cells treated with drugs were compared with those without drug treatment using a Mann-Whitney test.

We showed above that Iressa failed to abolish HER2 phosphorylation in surviving SKBR3 cells due to activation of alternative HER3 and HER4 receptors via the autocrine release of various ligands. Since Herceptin targets the HER2 receptor, we proceeded to investigate whether combined treatment of Herceptin with Iressa would abolish HER2 phosphorylation in SKBR3 cells. It has been shown that the combined treatment with Herceptin and Iressa in SKBR3 was either additive [22] or synergistic [33] in exerting anti-proliferative effects as well as having enhanced anti-tumour activity in BT-474 xenografts [12], [34]. The cell viability experiments confirmed that the combined treatment was more prominent in its anti-proliferative effect than either Iressa or Herceptin treatment alone (Figure 4A). FRET was used to assess the effect of combined treatment on HER2 phosphorylation in sensitive SKBR3 cells (Figure 4B). The assessment of HER2 phosphorylation by FRET showed that HER2 activation increased from basal levels during the first 2.5 days of combined Iressa and Herceptin (Figure 4B). However, after five days of treatment we observed a decrease of HER2 phosphorylation (Figure 4B) in concordance with a decrease of cell viability (Figure 4A). After seven days, there were too few surviving cells (Figure 4A) but the remaining surviving cells remain activated in HER2 (Figure 4B). These cells may represent resistant cells to combined treatment.

We hypothesized that the greater effect on cell viability with combined Iressa and Herceptin treatment must be due to greater EGFR suppression from adding Herceptin to Iressa treatment. This is illustrated by FRET experiments in EGFR phosphorylation (Figure 4C). Figure 4C shows the decrease of average lifetime of EGFR-Cy3b with pEGFR-Cy5 from 2.45 ns to 2.15 ns, indicating basal phosphorylation of EGFR in these cells. Treatment with 1 µM Iressa partially suppressed EGFR phosphorylation with an increase of the average lifetime of EGFR-Cy3b from 2.15 ns to 2.3 ns (p<0.001 compared to basal). The incomplete suppression of EGFR phosphorylation by Iressa may be explained by the compensatory increase in autocrine ligand release induced by Iressa shown previously. However, the combination of Iressa with Herceptin exerted greater suppression of EGFR phosphorylation (p<0.001 compared to basal) more than Iressa alone (Figure 4C). This result illustrates that the additive effect of combined therapy in the cell viability experiments (Figure 4A) was due to greater inhibition of EGFR phosphorylation with combined therapy.

In summary, a combined treatment of cells with Herceptin and Iressa exerts a greater suppression in EGFR and HER2 activation and induced an enhanced anti-proliferative effect.

## Discussion

The current literature has been inconsistent in its conclusion on the effects of TKIs on HER2 functions. Although there have been reports suggesting that TKIs inhibits HER2-driven signaling [22], [23], TKIs in fact do not fully inhibit HER2 oncogenic function at physiological doses [24]. Using FRET in single cell analysis we showed persistent HER2 phosphorylation in surviving TKIs treated cells. This does not contradict the current literature; rather the FRET analysis provides a novel sensitive insight beyond the present knowledge of the effects of TKIs on HER2 activation and other HER receptors. FRET may be sensitive enough to detect residue HER2 phosphorylation in single cells even when HER2 activation is below the detection limit of biochemical analysis for the whole cell lysate. The apparent difference from the current literature is also more an issue of different experimental conditions of EGFR inhibitor treatments. For example, in Moasser et al (2001), the experiments on HER2 phosphorylation were a function of Iressa dosage in SKBR3 cells [22]. HER2 phosphorylation was only minimally suppressed by 1 µM Iressa (we observed partial HER2 phosphorylation in some of the cells, Figure 1C) and only greatly reduced when the dose was increased to 10 µM [22]. We performed similar experiments but noted that 10 µM was toxic to cells. Therefore, the partial decrease in HER2 phosphorylation in Iressa treated SKBR3 cells is due to the effects of Iressa on EGFR/HER2 [22], [23] but we showed that the HER2 phosphorylation is not abolished in the surviving cells due to activation of HER2 via HER2/HER3 and HER2/HER4, mediated through autocrine ligand release.

EGFR TKI monotherapy results in a relatively poor response rate and the response is not usually sustained for the responders [5]. HER receptors are highly dynamic and the hierarchy of their activation changes with the availability of HER receptors and with drug treatment [21], [35]. For example, MCF-7 cells are not driven by HER2 over-expression and have a low level of EGFR. Yet when these cells are treated with an oestrogen deprivation anti-hormonal treatment such as tamoxifen, it has been shown that EGFR/HER2 heterodimer levels become elevated and autocrine loops are activated [35]. Iressa has been used to overcome hormone resistance in oestrogen deprived MCF-7 cells [35]. Thus, the response to these drugs may depend more on the activation status of HER receptors as well as their dimerisation partners, rather than the receptor concentration alone.

**Figure 5:**
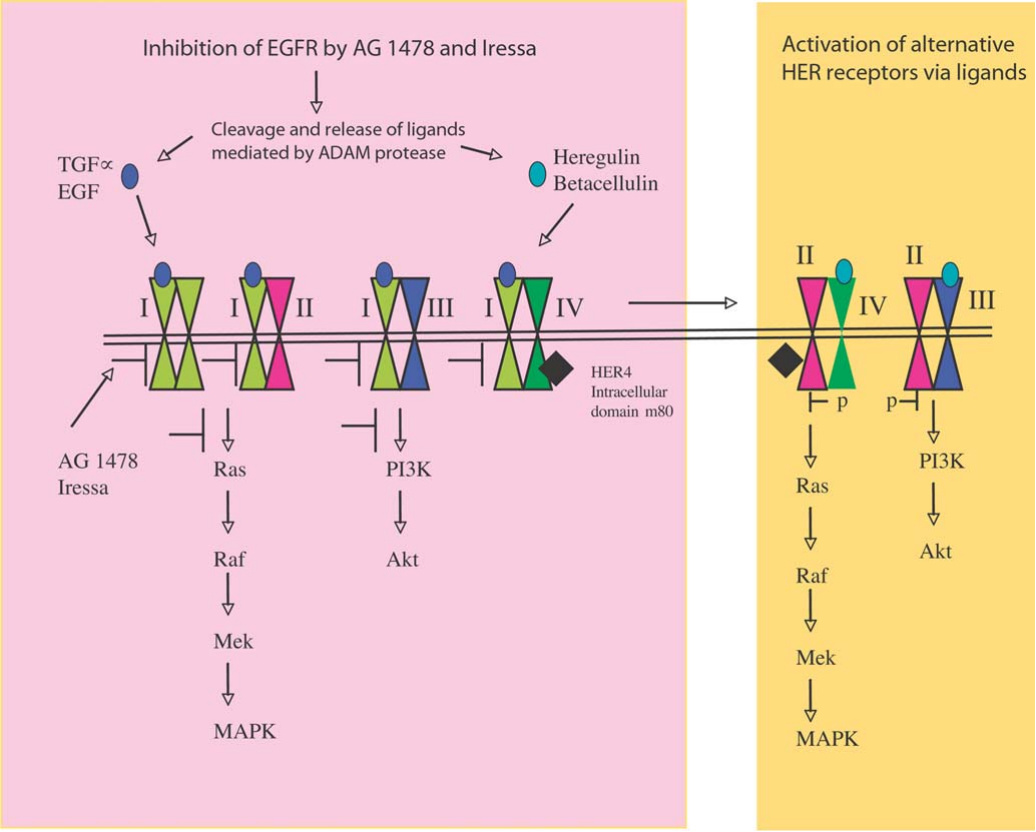
Mechanisms of resistance for tyrosine kinase inhibitors AG 1478 and Iressa. AG 1478 and Iressa treatment induce inactive EGFR homodimers and inactive EGFR/HER2 heterodimers. The treatment also decreases phopho-HER3 levels through inhibition of EGFR/HER3 with a decrease in PKB (Akt) activity. However, the treatment induces the autocrine release of various ligands including heregulin and betacellulin causing activation and cleavage of HER4, which in turn causes dimerization between HER2 and HER4. Following the initial decrease through inhibition of EGFR/HER3, phospho-HER3 as well as phospho-ERK1/2 and phospho-PKB (Ser473) activation augments again within 2 days of treatment due to the release of ligands causing dimerization between HER2/HER3 in addition to HER2/HER4.

Although it has been speculated that alternative HER receptor activation mediates resistance to targeted therapies, this is the first time that a molecular mechanism is provided to explain drug resistance in breast cancer cell lines. Quinazoline tyrosine kinase inhibitors of EGFR have been shown to induce inactive EGFR homodimers and EGFR/HER2 heterodimers in EGFR over-expressing cancer cells [36] as well as decreasing EGFR/HER3 mediated PI3K/Akt pathway [11]. However, here we showed that the inhibition of EGFR activation by AG 1478 and Iressa caused the release of various ligands including heregulin and betacellulin. The release of these ligands resulted in dimerisation of HER 2 and HER4, and proteolytic cleavage of HER4. Moreover, the heregulin release also reactivated HER3 via HER2/HER3 dimers along with downstream signalling pathways. These processes offer an explanation for resistance to Iressa. The model of resistance to Iressa is shown in Figure 5. The combined therapy of Herceptin and Iressa is additive in suppression of EGFR and HER2 activation as well as exerting its anti-proliferative effect, consistent with the report that combination of targeted therapies against both EGFR and HER2 is more effective that single agents in breast cancer [33].

The differential effect of AG 1478 and Iressa in inducing heregulin and betacellulin release is likely due to their different affinities and efficacies in the two cell lines. Therefore, AG 1478 and Iressa may produce a different ligand response in MCF-7 cells since Iressa has a higher affinity than AG 1478. Betacellulin is the ligand for EGFR/HER4 and heregulin is the ligand for HER3/HER4 and their release in response to drugs may be different. AG 1478 is less potent that Iressa in EGFR inhibition and thus produced a minimal betacellulin release.

In a paper by Zhou et al (2006) the authors found that among various genes examined in 44 different non-small cell lung cancer cell lines, only the expression of heregulin significantly correlated with insensitivity to Iressa [37]. Although HER3 expression was only very weakly correlated with Iressa sensitivity, the authors concluded that it is the heregulin-induced HER3 activation rather than the level causing insensitivity to Iressa [37]. We have shown that HER3 phosphorylation was suppressed by Iressa upon acute treatment in three breast cancer cell lines as well as A431 cells through suppression of EGFR/HER3 dimerization. However, the release of ligands (including heregulin and betacellulin) induced by Iressa treatment resulted in dimerization between HER4 and HER2 as well as HER3 and HER2. The effects of these dimerizations were the reactivation of phospho-HER3 and phospho-PKB (Ser473).

Sergina et al (2007) also observed the reactivation of phospho-HER3 with prolonged Iressa treatment [38]. The reactivation of HER3 may occur within several hours of Iressa treatment after the initial suppression of HER3 activation. The group explained that the reactivation of HER3 with prolonged Iressa treatment was due to a compensatory shift in the HER3 phosphorylation-dephosphorylation equilibrium as a result of increased HER3 expression and reduced phosphatase activity and concluded that “because HER3 signalling is buffered against an incomplete inhibition of HER2 kinase, much more potent TKIs or combination strategies are required to silence oncogenic HER2 signalling effectively” [38]. Our results confirmed the inability of TKIs to abolish HER2 phosphorylation in surviving cells due to activation of the alternative HER receptors (including that of HER2/HER3 and HER2/HER4) as a result of ligand release. Therefore, our results have contributed to the gaps in understanding the mechanisms of resistance to these targeted therapies.

Although exogenous heregulin enhanced aggregation [39] and increased invasiveness in breast cell lines [40], it has been reported to have an anti-proliferative effect [41] and thus may challenge the role of HER4 in mediating resistance to Iressa. Aguilar et al (1999) reported that some of the disparity on various effects of heregulin is due to variations in the cell lines, ligand dosage and the methodologies used between different investigators [42]. The group found no evidence that heregulin had any growth-inhibitory effects in human epithelial cells having used several different *in vitro* and *in vivo* assays in different cell lines. We have also shown that exogenous heregulin induced proliferation rather than exerting an anti-proliferative effect upon Iressa treatment, confirming the role of heregulin in mediating resistance to tyrosine kinase inhibitors of EGFR. Moreover, we confirmed the role of HER4 in mediating resistance to Iressa since anti-betacellulin antibody potentiated the anti-proliferative effect in combination with Iressa treatment.

Our results indicate how apparent targeted therapies for breast cancer patients have complex effects, offering treatment opportunities to overcome resistance in patients. It is anticipated that future therapy for breast cancer may involve targeting various HER receptors, their ligands [37] as well as metalloproteinases that mediate the cleavage of the ligands [43].

## Materials and Methods

### Materials and cell lines

A431, MCF-7, SKBR3 and MDAMB-453 cells were obtained from cell services at Cancer Research UK, Lincoln's Inn Fields (CR-UK). The cells were routinely cultured as monolayers in Dulbecco's modified eagle's medium (DMEM) supplemented with 7.5% (v/v) foetal bovine serum (FBS) at 37°C in a CO_2_ humidified atmosphere. Anti-HER2 antibody (recognize the intracellular residues surrounding Tyr1222), anti-phospho-HER2 antibody (Tyr1221/1222), anti-phospho-HER2 antibody (Tyr1248), anti-phospho-HER3 (Tyr1289), anti-HER4 antibody (recognize the intracellular residues near the carboxyl-terminus of human HER4) and anti-phosphotyrosine pTyr-100 were obtained from Cell Signalling Technology. F4-IgG1 mouse monoclonal antibody, (against residues 985–996 of the EGFR cytoplasmic domain) and FB2-IgG3 (monoclonal against phosphotyrosine) antibodies were obtained from the Monoclonal Antibody lab, Lincoln's Inn Fields. Antibodies recognizing PKB, phospho-PKB (Ser473), p44/42 MAP Kinase (Erk1/Erk2) and phospho-Erk1/Erk2 (Thr202/Tyr 204) were from Cell Signalling Technology. The monoclonal anti-β-actin and monoclonal anti-betacellulin were obtained from Sigma-Aldrich, USA. The rabbit anti-heregulin-1 precursor was obtained from Upstate, USA and recognizes amino acids 615–640 of the heregulin-1 precursor. The secondary goat anti-mouse IgG was purchased from Amersham Biosciences UK limited. AG 1478 a selective inhibitor of the EGFR tyrosine kinase (IC_50_ = 3 nM) was from Calbiochem UK. The mono-conjugated fluorophores Cy™3B and Cy5 were from Amersham Biosciences. Protein tyrosine phosphatase (YOP) from *Yersinia enterocolitica* (Recombinant, E.coli) was purchased from Calbiochem. Herceptin was courtesy of Genentech, and Iressa was given and granted permission to use in our experiments by Astrazeneca.

### Western blotting

The cells were grown to 80–100% confluency in a 6-well cell plate after seeding 30,000 cells. The cells were treated with different conditions as described. The cells were lysed in lysis buffer on ice for 30 minutes (Tris HCl, 20 mM; NaCl, 150 mM; NaF 100 mM; Na_4_P_2_0_7_ 10 mM; EDTA 10 mM with 1% Triton and protease inhibitor cocktail-Roche) and centrifuged at 4°C to remove of the insoluble cell pellets. Polyacrylamide gel electrophoresis was carried out employing 10 µg of protein in each lane. Western blots were performed using the primary antibodies mentioned above, at a 1∶1000 dilution. Antibodies were incubated overnight at 4°C. They were detected using a horseradish peroxidase-linked secondary antibody (a dilution of 1∶2000 goat anti-rabbit IgG) and visualized with an enhanced chemiluminescent (ECL) system (Amersham).

### Immunoprecipitation

MCF-7 and SKBR3 cells were grown to near confluency before lysis buffer as described above. The cell lysate was centrifuged for 5 minutes at maximum speed before transferring the supernatant to a new reaction vial. The supernatant was preabsorbed with prewashed Protein G Agarose beads (Roche) for 2 hours at 4°C after. The mixture of cell lysate and beads was centrifuged for 5 minutes at maximum speed before transferring the supernatant to a new reaction vial. Anti-HER4 was added (1∶100) to the supernatant and incubated overnight at 4°C. The next day, the immune-complex was collected by the addition of new beads and further incubation for 2 hours at 4°C. The beads were washed thoroughly with lysis buffer before boiling with 4× SDS. 40 µl was loaded per lane in SDS gel for western blot analysis.

### Cell Viability Experiments

Cells were grown in 24-well plates after seeding approximately 30,000 cells per well. The cells were grown for at least 24 hours before treatment with either 40 µg/ml Herceptin or 1 µM Iressa. For Iressa experiments, a DMSO control (1∶1000) was also performed. On the day of experiment, the cells were trypsinized and diluted with PBS. The viable cells were counted in a Cell Viability Analyzer (Vi-cell™ XR, Beckman Coulter) using Trypan blue to stain the dead cells.

### Förster Resonance Energy Transfer (FRET) measured by Fluorescence Lifetime Imaging Microscopy (FLIM)

FRET involves the transfer of energy from an excited donor molecule to a nearby (<7 nm) spectrally overlapping acceptor. FRET can be quantified by measuring fluorescence lifetime of the donor, which is reduced as energy is non-radiatively transferred via a dipole-dipole interaction. Spatial aspects of fluorescence lifetime may be assessed by using FLIM [44]. In this study we have monitored donor lifetime variations in the frequency (phase) domain where the excitation light is sinusoidally modulated at 80.218 MHz to excite the sample. The emitted light oscillates at the same modulation frequency but with a phase shift and a decrease in amplitude (demodulation). Determining these two parameters permits measurement of phase (τ_p_) and modulation depth (τ_m_) of the fluorescence. The lifetime, <τ>, is the average of phase shift and relative modulation depth (τ_m_+τ_p_)/2 of the emitted fluorescence signal [44], [45].

### Conjugation of donor and acceptor fluorophore to antibodies

F4 (anti-EGFR) and anti-HER2 were conjugated to Cy3b (donor fluorophore); FB2 (anti-phosphotyrosine as anti-pEGFR) and anti-phosphoHER2 were conjugated to Cy5 (acceptor fluorophore). 100 µl of N, N-Dimethylformamide (DMF) was added to 1 mg Cy3b to make a 10 mg/ml stock solution (15 mM). The 10 mg/ml stock of Cy3b was diluted in DMF 10 fold to 1 mg/ml (1.5 mM). 50 µl of this was added drop by drop into 450 µl antibody / 50 µl Bicine (1 M, pH 8) and conjugated as above. The final concentration of conjugated antibody with Cy3b was approximately 100 µg (150 µM). The solution was stirred in the dark for 1–2 hours. To conjugate FB2 (anti-phosphotyrosine as anti-pEGFR), anti-pHER2 with Cy5, 20 µl of DMF was added to a Cy5 vial. FB2 dye in DMF was then added drop by drop to 450 µl antibody / 50 µl Bicine (1 M, pH 8) while stirring. The solution was stirred in the dark for 1–2 hours. The conjugated antibodies were separated from free dyes by column chromatography. The dye/protein (D/P) ratios were maintained constant per experiment. The D/P ratios were measured by UV/visible spectroscopy at 280 nm to determine antibodies' concentrations. The concentration of F4-Cy3b and anti-HER2-Cy3b were detected at 552 nm and FB2-Cy5 and anti-pHER2-Cy5 at 650 nm. The D/P ratios were calculated using the protocol provided by Amersham Biosciences for Cy™3B mono-reactive dye:
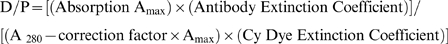



### FRET Experiments

Cells were grown in 24-well plates on cover slips after seeding 15,000 cells per well. For Herceptin and Iressa experiments, the cells were left to grow for at least 24 hours before treatment with drugs. For growth factor experiments, cells were treated with 50 ng/ml of EGF, 100 ng/ml of heregulin β and heregulin-β1 for 10 minutes following serum starvation of 16 hours. Following stimulation, the cells were fixed with 4% paraformaldehyde (PFA) at room temp for 10 minutes. 500 µl of 0.2% (v/v) Triton X-100 was added per well for 5 minutes to make the cell membrane permeable followed by 1 mg/ml fresh sodium borohydrate / PBS for 10 minutes to quench background fluorescence. Between each of these steps, the cells were washed three times with PBS. The cells were blocked with 1% w/v BSA / PBS for 1 hour. For experiments involving the protein tyrosine phosphatase from *Yersinia enterocolitica* (YOP), 50 units of phosphatase in 50 µl reaction buffer (50 mM Tris-HCL, pH 7.2, 150 mM NaCl, 5 mM DTT, 2.5 Mm Na_2_EDTA, and 100 µg/ml BSA) was used for each coverslip after fixing with PFA.

After blocking the cells were incubated with conjugated donor antibodies (anti-EGFR-Cy3b or anti-HER2-Cy3b) for 2 hours. For cells that required detection with the acceptor fluorophore, a further incubation with either FB2-Cy5 or anti-pHER2-Cy5 for 2 hours took place to assess EGFR and HER2 phosphorylation states respectively. The cover slips mounted on the slides with Mowiol mounting medium containing 2.5% (w/v) 1,4-diazabicyclo (2.2.2) octane as an anti-fade. The slides were left at 37°C, in an incubator for 1 hour and at room temperature overnight prior to image acquisition.

For FRET experiments, all images were taken using a Zeiss Plan-APOCHROMAT ×100/1.4 NA phase 3-oil objective with images recorded at a modulation frequency of 80.218 MHz. The donor (anti-EGFR-Cy3b or anti-HER2-Cy3b) was excited using 514-nm line of an argon/krypton laser, and the resultant fluorescence was separated using a combination of dichroic beam splitter (Q565 LP; CHROMA technology Corp.) and narrow band emitter filter (BP 610 /75; Lys and Optik).

### FRET Data Interpretation and Statistical analysis

The Average lifetime of the donor fluorophore (anti-EGFR-Cy3b and anti-HER2-Cy3b) from each condition was shown as scatter diagrams. The basal condition was defined as the basal phosphorylation of the HER receptor, indicated by the decrease of lifetime of the donor in the presence of the acceptor without growth factor stimulation or drug treatment. The basal phosphorylation was due to autocrine signalling pathways of the cancer cells as a result of ligand stimulation, e.g. basal EGFR phosphorylation due to autocrine receptor activation in A431 cells [46]. The enhanced decrease in the average lifetime indicated further phosphorylation of the receptor due to dimerization with its partners. In each experiment (minimum of 3 experiments), the lifetimes of a minimum 5 cells or groups of cells were obtained and medians of these measurements were displayed in the scatter diagram. A Mann-Whitney test was used to compare the medians of the average lifetime between the basal condition and those stimulated with ligands or treated with drugs.

## Supporting Information

Figure S1 Inhibition of EGFR with TKI AG 1478 does not abolish HER2 phosphorylation. A, A431, MCF-7, MDAMB-453 and SKBR3 cells were grown to near confluency before lysis for western blot analysis. The membrane was probed with either anti-HER2 or anti-EGFR antibody. B, A431 cells pre-treated with increasing doses of AG 1478 for two hours before being stimulated with 100 ng/ml EGF for 10 minutes. The cells were assessed for HER2 phosphorylation by FRET. C, A431 cells were pre-treated by increasing doses of AG 1478 as illustrated before 100 ng/ml EGF stimulation and western blot analysis. The phosphorylation of PKB on Ser473 and Erk1/Erk2 (p44/42 MAP Kinase) on Thr202/Tyr204 was determined using phosphospecific antibodies. The total endogenous levels of Erk1/Erk2 were assessed by western blot using anti-ERK antibodies. D, Upper panels, A431 cells and two other breast cancer cell lines MDAMB-453 and SKBR3 cells were assessed for HER2 phosphorylation after pre-treatment of the cells with 3 µM AG 1478 for two hours. Lower panels, A431, MDAMB-453 and SKBR3 cells were lysed for western blot analysis after treatment with either 3 µM AG 1478 or vehicle for two hours. The phosphorylation of HER2, phosphoPKB Ser473 and Erk1/Erk2 was determined using phosphospecific antibodies
(4.75 MB EPS)
